# *N*,*N*′-Bis[(1*H*-imidazol-1-yl)meth­yl]-2,2′-(disulfanedi­yl)dianiline

**DOI:** 10.1107/S1600536812025330

**Published:** 2012-06-13

**Authors:** Hon Man Lee, Chang-Chih Hsieh, Yih-Chern Horng

**Affiliations:** a National Changhua University of Education, Department of Chemistry, Changhua, Taiwan 50058

## Abstract

The symmetrical title compound, C_20_H_20_N_6_S_2_, contains a disulfide bond of 2.0884 (6) Å. The C—S—S—C torsion angle is −59.57 (7)°. In the crystal, classical N—H⋯N and non-classical C—H⋯N hydrogen bonds link the compounds into chains along the *a* axis.

## Related literature

For transition metal complexes having related ligands, see: Hsieh *et al.* (2009*a* ▶,*b* ▶).
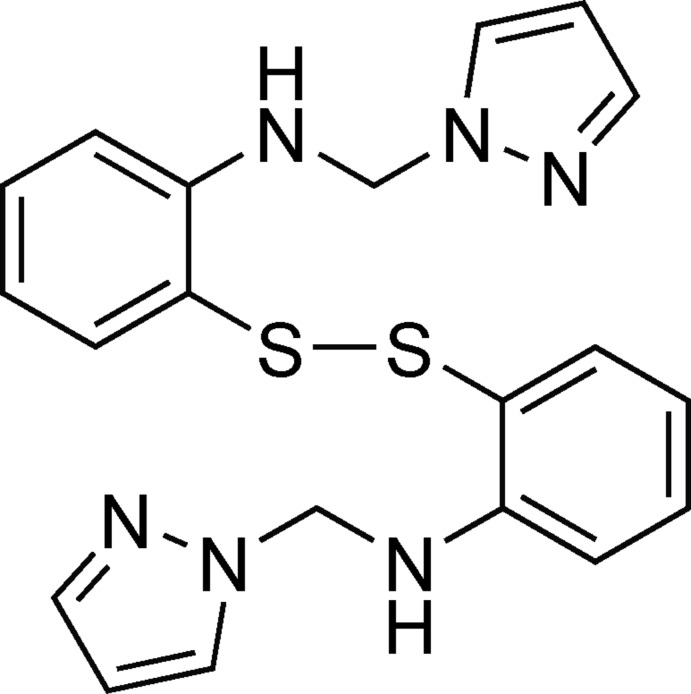


## Experimental

### 

#### Crystal data

C_20_H_20_N_6_S_2_
*M* *_r_* = 408.56
Monoclinic, 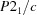

*a* = 11.2009 (10) Å
*b* = 11.6067 (10) Å
*c* = 15.5230 (14) Å
β = 97.162 (2)°
*V* = 2002.3 (3) Å^3^
*Z* = 4
Mo *K*α radiation
μ = 0.28 mm^−1^
*T* = 150 K
0.38 × 0.32 × 0.24 mm

#### Data collection

Bruker SMART APEXII diffractometer
Absorption correction: multi-scan (*SADABS*; Sheldrick, 2003 ▶) *T*_min_ = 0.888, *T*_max_ = 0.954
23330 measured reflections
4970 independent reflections
3844 reflections with *I* > 2σ(*I*)
*R*_int_ = 0.026

#### Refinement

*R*[*F* ^2^ > 2σ(*F* ^2^)] = 0.033
*wR*(*F* ^2^) = 0.128
*S* = 0.96
4970 reflections
261 parameters
H atoms treated by a mixture of independent and constrained refinement
Δρ_max_ = 0.27 e Å^−3^
Δρ_min_ = −0.26 e Å^−3^

### 

Data collection: *APEX2* (Bruker, 2007 ▶); cell refinement: *SAINT* (Bruker, 2007 ▶); data reduction: *SAINT*; program(s) used to solve structure: *SHELXTL* (Sheldrick, 2008 ▶); program(s) used to refine structure: *SHELXTL*; molecular graphics: *SHELXTL*; software used to prepare material for publication: *DIAMOND* (Brandenburg, 2006 ▶).

## Supplementary Material

Crystal structure: contains datablock(s) I, global. DOI: 10.1107/S1600536812025330/wn2478sup1.cif

Structure factors: contains datablock(s) I. DOI: 10.1107/S1600536812025330/wn2478Isup2.hkl

Supplementary material file. DOI: 10.1107/S1600536812025330/wn2478Isup3.cml

Additional supplementary materials:  crystallographic information; 3D view; checkCIF report

## Figures and Tables

**Table 1:** Hydrogen-bond geometry (Å, °)

*D*—H⋯*A*	*D*—H	H⋯*A*	*D*⋯*A*	*D*—H⋯*A*
N6—H22⋯N4^i^	0.818 (17)	2.252 (17)	2.9825 (19)	148.9 (15)
C1—H1⋯N2^ii^	0.93	2.58	3.448 (2)	155

Symmetry codes: (i) 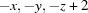; (ii) 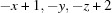.

**Fig. 1.**
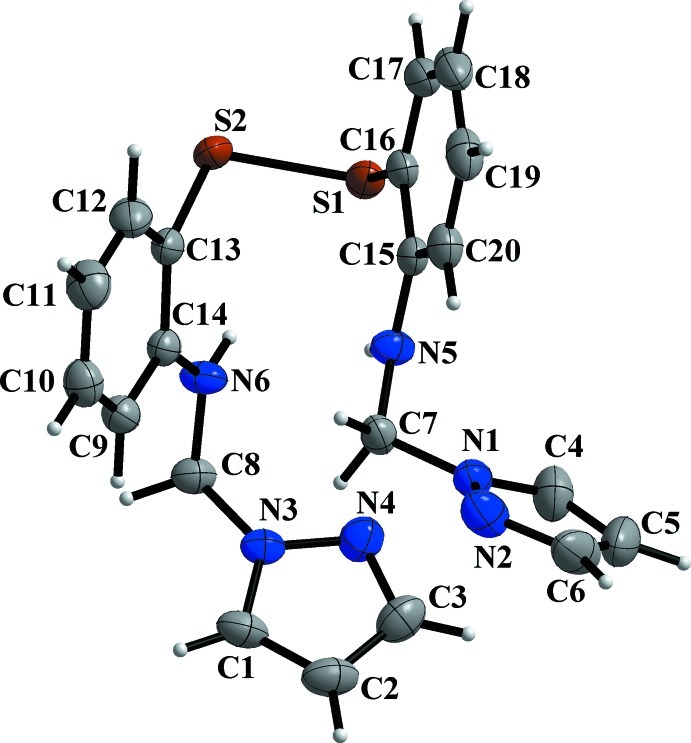
The structure of the title compound, showing 50% probability displacement ellipsoids for the non-hydrogen atoms. The H atoms are depicted as spheres of arbitrary radius.

**Fig. 2.**
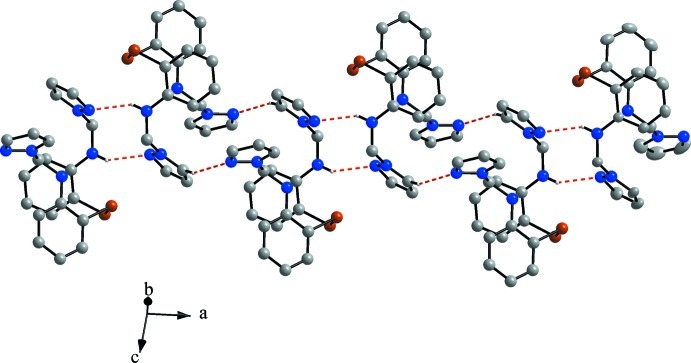
A view of the one-dimensional hydrogen-bonded chain, showing the hydrogen bonds as dashed lines.

## supplementary crystallographic information

### Comment

In our research, we have focused on the synthesis of low-molecular-weight complexes, which exhibited the structural and functional features of the active sites in particular enzymes, such as Ni-containing methyl coenzyme *M* reductase (Hsieh *et al.,* 2009*b*) and Fe-containing nitrile hydratase (Hsieh *et al.,* 2009*a*). The synthesis and crystal structure determination of the title compound are reported here. This compound will be used as a coordination ligand in related studies.

The disulfide bond length is 2.0884 (6) Å and the C—S—S—C torsion angle is 59.56 (7)° (Fig. 1). In the crystal structure the compound does not exhibit crystallographic symmetry. Classical N—H···N and non-classical C—H···N hydrogen bonds (Table 1) link the compounds into one-dimensional chains along the *a*-axis (Fig. 2).

### Experimental

Pyrazole (1.0 g, 14.7 mmol) was dissolved in 50 ml CH_2_Cl_2_ and treated with formaldehyde (37%, 1.19 g, 14.7 mmol). After stirring at room temperature for 10 min, the mixture was added to a batch of 2-aminophenyl disulfide (1.82 g, 7.32 mmol) and stirred for a further 12 h. After completion of the reaction, the solution was extracted with distilled water. Portions of the CH_2_Cl_2_ extract were collected and dried using anhydrous MgSO_4_. The solvent was then removed under vacuum to afford a yellow powder (2.56 g, 86%). Crystals suitable for X-ray diffraction analysis were obtained by slow evaporation of a tetrahydrofuran solution of the compound at -4 °C.

### Refinement

C-bound H atoms were positioned geometrically and refined as riding, with C_aryl_—H = 0.93 and C_methylene_—H = 0.97 Å; *U*_iso_(H) = 1.2*U*_eq_(C). N-bound H atoms were located in a difference Fourier map and freely refined (N5—H21 = 0.833 (17) Å and N6—H22 = 0.818 (17) Å.

### Crystal data

**Table:**

C_20_H_20_N_6_S_2_	*F*(000) = 840
*M**_r_* = 408.56	*D*_x_ = 1.355 Mg m^−^^3^
Monoclinic, *P*2_1_/*c*	Mo *K*α radiation, λ = 0.71073 Å
*a* = 11.2009 (10) Å	Cell parameters from 6226 reflections
*b* = 11.6067 (10) Å	θ = 2.2–27.2°
*c* = 15.5230 (14) Å	µ = 0.28 mm^−^^1^
β = 97.162 (2)°	*T* = 150 K
*V* = 2002.3 (3) Å^3^	Block, yellow
*Z* = 4	0.38 × 0.32 × 0.24 mm

### Data collection

**Table:**

Bruker SMART APEXII diffractometer	4970 independent reflections
Radiation source: fine-focus sealed tube	3844 reflections with *I* > 2σ(*I*)
Graphite monochromator	*R*_int_ = 0.026
ω scans	θ_max_ = 28.3°, θ_min_ = 1.8°
Absorption correction: multi-scan (*SADABS*; Sheldrick, 2003)	*h* = −14→14
*T*_min_ = 0.888, *T*_max_ = 0.954	*k* = −15→15
23330 measured reflections	*l* = −20→20

### Refinement

**Table:**

Refinement on *F*^2^	Primary atom site location: structure-invariant direct methods
Least-squares matrix: full	Secondary atom site location: difference Fourier map
*R*[*F*^2^ > 2σ(*F*^2^)] = 0.033	Hydrogen site location: inferred from neighbouring sites
*wR*(*F*^2^) = 0.128	H atoms treated by a mixture of independent and constrained refinement
*S* = 0.96	*w* = 1/[σ^2^(*F*_o_^2^) + (0.1*P*)^2^ + 0.0208*P*] where *P* = (*F*_o_^2^ + 2*F*_c_^2^)/3
4970 reflections	(Δ/σ)_max_ < 0.001
261 parameters	Δρ_max_ = 0.27 e Å^−^^3^
0 restraints	Δρ_min_ = −0.26 e Å^−^^3^

### Special details

**Table:**

Geometry. All e.s.d.'s (except the e.s.d. in the dihedral angle between two l.s. planes) are estimated using the full covariance matrix. The cell e.s.d.'s are taken into account individually in the estimation of e.s.d.'s in distances, angles and torsion angles; correlations between e.s.d.'s in cell parameters are only used when they are defined by crystal symmetry. An approximate (isotropic) treatment of cell e.s.d.'s is used for estimating e.s.d.'s involving l.s. planes.
Refinement. Refinement of *F*^2^ against ALL reflections. The weighted *R*-factor *wR* and goodness of fit *S* are based on *F*^2^, conventional *R*-factors *R* are based on *F*, with *F* set to zero for negative *F*^2^. The threshold expression of *F*^2^ > σ(*F*^2^) is used only for calculating *R*-factors(gt) *etc*. and is not relevant to the choice of reflections for refinement. *R*-factors based on *F*^2^ are statistically about twice as large as those based on *F*, and *R*- factors based on ALL data will be even larger.

### Fractional atomic coordinates and isotropic or equivalent isotropic displacement parameters (Å^2^)

**Table:**

	*x*	*y*	*z*	*U*_iso_*/*U*_eq_	
S1	−0.02094 (3)	0.08013 (3)	0.76606 (3)	0.03264 (13)	
S2	0.01131 (3)	0.25438 (3)	0.79328 (3)	0.03404 (13)	
N3	0.20015 (10)	0.01306 (11)	1.07381 (8)	0.0302 (3)	
N1	0.36181 (11)	−0.17778 (11)	0.86130 (8)	0.0317 (3)	
N4	0.14835 (12)	−0.08760 (11)	1.04385 (10)	0.0390 (3)	
N2	0.47440 (12)	−0.20760 (14)	0.84774 (10)	0.0460 (4)	
N6	0.10991 (11)	0.15689 (12)	0.97273 (8)	0.0338 (3)	
N5	0.21637 (11)	−0.02980 (11)	0.82527 (8)	0.0312 (3)	
C7	0.33157 (12)	−0.05670 (13)	0.87099 (10)	0.0309 (3)	
H7A	0.3316	−0.0392	0.9321	0.037*	
H7B	0.3923	−0.0091	0.8491	0.037*	
C8	0.13251 (13)	0.12062 (13)	1.06103 (10)	0.0321 (3)	
H8A	0.0561	0.1112	1.0836	0.039*	
H8B	0.1768	0.1808	1.0946	0.039*	
C1	0.30946 (13)	−0.00488 (16)	1.12009 (10)	0.0384 (4)	
H1	0.3605	0.0512	1.1469	0.046*	
C3	0.22890 (16)	−0.16789 (15)	1.07264 (12)	0.0446 (4)	
H3	0.2182	−0.2463	1.0622	0.053*	
C2	0.33048 (15)	−0.12061 (16)	1.12002 (11)	0.0455 (4)	
H2	0.3982	−0.1594	1.1461	0.055*	
C4	0.29052 (19)	−0.26996 (15)	0.86760 (14)	0.0490 (4)	
H4	0.2098	−0.2687	0.8762	0.059*	
C5	0.3583 (2)	−0.36500 (17)	0.85913 (13)	0.0607 (6)	
H5	0.3348	−0.4417	0.8613	0.073*	
C6	0.4712 (2)	−0.32236 (18)	0.84645 (13)	0.0595 (6)	
H6	0.5365	−0.3686	0.8381	0.071*	
C13	0.16083 (13)	0.25746 (12)	0.84587 (10)	0.0288 (3)	
C15	0.19876 (12)	−0.00747 (12)	0.73713 (9)	0.0277 (3)	
C14	0.19334 (12)	0.21299 (12)	0.93032 (9)	0.0271 (3)	
C16	0.09108 (13)	0.04528 (12)	0.69947 (10)	0.0304 (3)	
C9	0.31319 (13)	0.22646 (13)	0.96785 (10)	0.0317 (3)	
H9	0.3368	0.1982	1.0234	0.038*	
C10	0.39688 (14)	0.28116 (14)	0.92343 (11)	0.0372 (4)	
H10	0.4754	0.2902	0.9500	0.045*	
C11	0.36564 (16)	0.32242 (15)	0.84051 (12)	0.0429 (4)	
H11	0.4225	0.3578	0.8105	0.052*	
C20	0.28490 (14)	−0.03762 (13)	0.68293 (10)	0.0342 (3)	
H20	0.3558	−0.0736	0.7063	0.041*	
C17	0.07364 (16)	0.06746 (13)	0.61034 (11)	0.0389 (4)	
H17	0.0026	0.1023	0.5859	0.047*	
C18	0.15999 (17)	0.03861 (14)	0.55792 (11)	0.0433 (4)	
H18	0.1478	0.0544	0.4987	0.052*	
C19	0.26499 (16)	−0.01419 (14)	0.59480 (11)	0.0415 (4)	
H19	0.3232	−0.0343	0.5597	0.050*	
C12	0.24702 (15)	0.31009 (14)	0.80253 (10)	0.0382 (4)	
H12	0.2251	0.3379	0.7466	0.046*	
H22	0.0410 (15)	0.1489 (14)	0.9491 (11)	0.029 (4)*	
H21	0.1641 (15)	−0.0085 (15)	0.8556 (11)	0.036 (5)*	

### Atomic displacement parameters (Å^2^)

**Table:**

	*U*^11^	*U*^22^	*U*^33^	*U*^12^	*U*^13^	*U*^23^
S1	0.02746 (19)	0.0298 (2)	0.0386 (2)	0.00133 (13)	−0.00418 (14)	0.00020 (15)
S2	0.0400 (2)	0.0265 (2)	0.0330 (2)	0.00832 (14)	−0.00588 (16)	0.00215 (14)
N3	0.0269 (6)	0.0364 (7)	0.0274 (6)	0.0039 (5)	0.0033 (5)	0.0034 (5)
N1	0.0289 (6)	0.0306 (7)	0.0343 (7)	0.0044 (5)	−0.0016 (5)	−0.0033 (5)
N4	0.0324 (7)	0.0366 (7)	0.0478 (8)	0.0018 (5)	0.0038 (6)	0.0016 (6)
N2	0.0330 (7)	0.0521 (9)	0.0504 (9)	0.0144 (6)	−0.0048 (6)	−0.0143 (7)
N6	0.0277 (6)	0.0436 (8)	0.0283 (7)	−0.0016 (5)	−0.0029 (5)	0.0089 (5)
N5	0.0277 (6)	0.0371 (7)	0.0282 (6)	0.0087 (5)	0.0018 (5)	0.0003 (5)
C7	0.0282 (7)	0.0285 (7)	0.0346 (8)	0.0019 (5)	−0.0018 (6)	−0.0058 (6)
C8	0.0311 (7)	0.0373 (8)	0.0278 (7)	0.0064 (6)	0.0034 (6)	0.0037 (6)
C1	0.0323 (7)	0.0538 (10)	0.0279 (8)	0.0103 (7)	−0.0009 (6)	0.0002 (7)
C3	0.0440 (9)	0.0375 (9)	0.0533 (11)	0.0108 (7)	0.0104 (8)	0.0060 (8)
C2	0.0422 (9)	0.0565 (11)	0.0370 (9)	0.0210 (8)	0.0023 (7)	0.0054 (8)
C4	0.0564 (11)	0.0355 (9)	0.0553 (12)	−0.0075 (8)	0.0083 (9)	−0.0083 (8)
C5	0.0962 (17)	0.0302 (9)	0.0521 (12)	0.0047 (10)	−0.0055 (11)	−0.0059 (8)
C6	0.0685 (13)	0.0504 (11)	0.0531 (11)	0.0337 (10)	−0.0175 (10)	−0.0168 (9)
C13	0.0360 (7)	0.0219 (7)	0.0275 (7)	0.0029 (5)	0.0001 (6)	−0.0020 (5)
C15	0.0316 (7)	0.0211 (7)	0.0296 (7)	−0.0043 (5)	0.0005 (5)	−0.0017 (5)
C14	0.0308 (7)	0.0230 (7)	0.0272 (7)	0.0027 (5)	0.0025 (5)	−0.0017 (5)
C16	0.0340 (7)	0.0235 (7)	0.0318 (8)	−0.0052 (5)	−0.0033 (6)	−0.0024 (6)
C9	0.0328 (7)	0.0318 (8)	0.0295 (8)	0.0011 (6)	0.0006 (6)	−0.0012 (6)
C10	0.0331 (8)	0.0364 (8)	0.0422 (9)	−0.0037 (6)	0.0050 (6)	−0.0063 (7)
C11	0.0450 (9)	0.0420 (10)	0.0435 (10)	−0.0103 (7)	0.0125 (7)	0.0004 (7)
C20	0.0358 (7)	0.0285 (8)	0.0385 (9)	−0.0057 (6)	0.0057 (6)	−0.0043 (6)
C17	0.0489 (9)	0.0294 (8)	0.0349 (9)	−0.0063 (7)	−0.0089 (7)	0.0011 (6)
C18	0.0642 (11)	0.0355 (9)	0.0290 (8)	−0.0150 (8)	0.0010 (7)	−0.0001 (7)
C19	0.0542 (10)	0.0348 (8)	0.0378 (9)	−0.0143 (7)	0.0146 (7)	−0.0071 (7)
C12	0.0510 (9)	0.0346 (8)	0.0289 (8)	−0.0041 (7)	0.0051 (7)	0.0033 (6)

### Geometric parameters (Å, º)

**Table:**

S1—C16	1.7691 (16)	C4—C5	1.355 (3)
S1—S2	2.0884 (6)	C4—H4	0.9300
S2—C13	1.7697 (15)	C5—C6	1.394 (3)
N3—C1	1.3562 (18)	C5—H5	0.9300
N3—N4	1.3605 (18)	C6—H6	0.9300
N3—C8	1.4609 (18)	C13—C12	1.385 (2)
N1—C4	1.346 (2)	C13—C14	1.414 (2)
N1—N2	1.3493 (18)	C15—C20	1.401 (2)
N1—C7	1.4577 (19)	C15—C16	1.4127 (19)
N4—C3	1.335 (2)	C14—C9	1.4039 (19)
N2—C6	1.333 (3)	C16—C17	1.397 (2)
N6—C14	1.3727 (19)	C9—C10	1.385 (2)
N6—C8	1.4260 (19)	C9—H9	0.9300
N6—H22	0.818 (17)	C10—C11	1.377 (3)
N5—C15	1.3823 (18)	C10—H10	0.9300
N5—C7	1.4276 (17)	C11—C12	1.392 (2)
N5—H21	0.833 (17)	C11—H11	0.9300
C7—H7A	0.9700	C20—C19	1.385 (2)
C7—H7B	0.9700	C20—H20	0.9300
C8—H8A	0.9700	C17—C18	1.381 (3)
C8—H8B	0.9700	C17—H17	0.9300
C1—C2	1.364 (2)	C18—C19	1.385 (3)
C1—H1	0.9300	C18—H18	0.9300
C3—C2	1.389 (3)	C19—H19	0.9300
C3—H3	0.9300	C12—H12	0.9300
C2—H2	0.9300		
			
C16—S1—S2	102.84 (5)	C4—C5—H5	127.6
C13—S2—S1	104.05 (5)	C6—C5—H5	127.6
C1—N3—N4	111.56 (13)	N2—C6—C5	112.10 (16)
C1—N3—C8	128.45 (14)	N2—C6—H6	123.9
N4—N3—C8	119.71 (12)	C5—C6—H6	123.9
C4—N1—N2	112.46 (15)	C12—C13—C14	119.78 (14)
C4—N1—C7	127.68 (14)	C12—C13—S2	117.49 (12)
N2—N1—C7	119.81 (13)	C14—C13—S2	122.68 (11)
C3—N4—N3	104.09 (14)	N5—C15—C20	121.57 (13)
C6—N2—N1	103.56 (15)	N5—C15—C16	120.00 (13)
C14—N6—C8	123.45 (13)	C20—C15—C16	118.43 (14)
C14—N6—H22	120.0 (11)	N6—C14—C9	121.79 (13)
C8—N6—H22	116.2 (11)	N6—C14—C13	120.39 (13)
C15—N5—C7	122.80 (13)	C9—C14—C13	117.81 (13)
C15—N5—H21	118.7 (12)	C17—C16—C15	119.77 (14)
C7—N5—H21	116.1 (12)	C17—C16—S1	120.99 (12)
N5—C7—N1	111.52 (12)	C15—C16—S1	119.24 (11)
N5—C7—H7A	109.3	C10—C9—C14	121.06 (14)
N1—C7—H7A	109.3	C10—C9—H9	119.5
N5—C7—H7B	109.3	C14—C9—H9	119.5
N1—C7—H7B	109.3	C11—C10—C9	121.06 (15)
H7A—C7—H7B	108.0	C11—C10—H10	119.5
N6—C8—N3	114.18 (12)	C9—C10—H10	119.5
N6—C8—H8A	108.7	C10—C11—C12	118.55 (15)
N3—C8—H8A	108.7	C10—C11—H11	120.7
N6—C8—H8B	108.7	C12—C11—H11	120.7
N3—C8—H8B	108.7	C19—C20—C15	120.38 (15)
H8A—C8—H8B	107.6	C19—C20—H20	119.8
N3—C1—C2	107.18 (15)	C15—C20—H20	119.8
N3—C1—H1	126.4	C18—C17—C16	121.16 (16)
C2—C1—H1	126.4	C18—C17—H17	119.4
N4—C3—C2	112.12 (16)	C16—C17—H17	119.4
N4—C3—H3	123.9	C17—C18—C19	119.00 (15)
C2—C3—H3	123.9	C17—C18—H18	120.5
C1—C2—C3	105.04 (14)	C19—C18—H18	120.5
C1—C2—H2	127.5	C18—C19—C20	121.26 (16)
C3—C2—H2	127.5	C18—C19—H19	119.4
N1—C4—C5	107.17 (19)	C20—C19—H19	119.4
N1—C4—H4	126.4	C13—C12—C11	121.72 (15)
C5—C4—H4	126.4	C13—C12—H12	119.1
C4—C5—C6	104.70 (18)	C11—C12—H12	119.1
			
C16—S1—S2—C13	−59.57 (8)	C8—N6—C14—C13	−173.15 (14)
C1—N3—N4—C3	−0.24 (18)	C12—C13—C14—N6	−177.59 (14)
C8—N3—N4—C3	−174.71 (13)	S2—C13—C14—N6	5.04 (19)
C4—N1—N2—C6	−0.47 (19)	C12—C13—C14—C9	1.3 (2)
C7—N1—N2—C6	177.01 (14)	S2—C13—C14—C9	−176.10 (10)
C15—N5—C7—N1	−81.64 (17)	N5—C15—C16—C17	−179.88 (13)
C4—N1—C7—N5	−44.9 (2)	C20—C15—C16—C17	−0.9 (2)
N2—N1—C7—N5	138.08 (14)	N5—C15—C16—S1	−0.91 (19)
C14—N6—C8—N3	−80.33 (18)	C20—C15—C16—S1	178.09 (10)
C1—N3—C8—N6	118.43 (16)	S2—S1—C16—C17	−83.60 (12)
N4—N3—C8—N6	−68.13 (18)	S2—S1—C16—C15	97.44 (11)
N4—N3—C1—C2	0.44 (18)	N6—C14—C9—C10	178.62 (14)
C8—N3—C1—C2	174.31 (14)	C13—C14—C9—C10	−0.2 (2)
N3—N4—C3—C2	−0.06 (19)	C14—C9—C10—C11	−1.1 (2)
N3—C1—C2—C3	−0.44 (18)	C9—C10—C11—C12	1.3 (2)
N4—C3—C2—C1	0.3 (2)	N5—C15—C20—C19	−179.97 (14)
N2—N1—C4—C5	0.8 (2)	C16—C15—C20—C19	1.0 (2)
C7—N1—C4—C5	−176.40 (15)	C15—C16—C17—C18	0.1 (2)
N1—C4—C5—C6	−0.8 (2)	S1—C16—C17—C18	−178.89 (12)
N1—N2—C6—C5	−0.1 (2)	C16—C17—C18—C19	0.6 (2)
C4—C5—C6—N2	0.6 (2)	C17—C18—C19—C20	−0.4 (2)
S1—S2—C13—C12	112.56 (11)	C15—C20—C19—C18	−0.4 (2)
S1—S2—C13—C14	−70.02 (12)	C14—C13—C12—C11	−1.1 (2)
C7—N5—C15—C20	16.4 (2)	S2—C13—C12—C11	176.41 (13)
C7—N5—C15—C16	−164.62 (13)	C10—C11—C12—C13	−0.2 (3)
C8—N6—C14—C9	8.0 (2)		

### Hydrogen-bond geometry (Å, º)

**Table:**

*D*—H···*A*	*D*—H	H···*A*	*D*···*A*	*D*—H···*A*
N6—H22···N4^i^	0.818 (17)	2.252 (17)	2.9825 (19)	148.9 (15)
C1—H1···N2^ii^	0.93	2.58	3.448 (2)	155

Symmetry codes: (i) −*x*, −*y*, −*z*+2; (ii) −*x*+1, −*y*, −*z*+2.
